# Reliability of Hand-Held Dynamometer for assessing Isometric Lumbar Muscles Strength in Asymptomatic Healthy Population

**DOI:** 10.12669/pjms.37.2.3621

**Published:** 2021

**Authors:** Fahad Tanveer, Syed Asadullah Arslan, Haider Darain, Ashfaq Ahmad

**Affiliations:** 1 Dr. Fahad Tanveer, PhD (Scholar) University Institute of Physical Therapy, The University of Lahore, Lahore, Pakistan; 2 Dr. Syed Asadullah Arslan, PhD University Institute of Physical Therapy, The University of Lahore, Lahore, Pakistan; 3 Dr. Haider Darain, PhD, Institute of Physical Medicine & Rehabilitation, Khyber Medical University, Peshawar, Pakistan; 4 Dr. Ashfaq Ahmad, PhD University Institute of Physical Therapy, The University of Lahore, Lahore, Pakistan

**Keywords:** Lumbar region, Muscle strength dynamometer, Reliability

## Abstract

**Objective:**

To determine intra-rater and inter-rater reliability of hand-held dynamometer for assessing isometric lumbar muscle strength in asymptomatic healthy population.

**Methods:**

It was a cross-sectional study conducted at the department of physiotherapy, University of Lahore Teaching Hospital, Lahore, Pakistan, from July 2020 to August 2020 through non probability-purposive sampling technique. Thirty healthy subjects were tested at thirty-degree lumbar flexion and zero-degree lumbar extension positions. Two raters assessed isometric strength of lumbar flexor and extensor muscles, by a hand-held dynamometer. Strength was measured and recorded by each of the two raters and re-assessed after a week. Correlation and pairwise comparison were done between readings. ICC values were calculated for the assessment of isometric lumbar muscle strength using handheld dynamometer.

**Results:**

A total of 30 healthy subjects had participated with mean age of 22.84±1.21 years, height 174.33±6.83 cm, weight 68.58±5.08 kg and BMI 22.52±0.35. Findings showed an excellent intra-rater (ICC 2, k = 0.95 to 0.97) and inter-rater (ICC 2, k = 0.94 to 0.95) reliability.

**Conclusions:**

Hand held dynamometer demonstrated an excellent intra- and inter-rater reliability for assessment of isometric lumbar muscles strength of healthy subject at clinical setting as it is simple to use, portable and cost-effective for the precise measurement of lumbar muscles strength.

## INTRODUCTION

Lumbar muscles strength plays a vital role in the physical performance and routine physical activity. These lumbar muscles provide lumbar stability during functional movements, transfer and control.^1^ On the other hand, decreased lumbar strength can trigger the surrounding muscles to become hypertonic^2^ which lead to low back pain^3^ and an estimated risk of injury which eventually impact activities of daily living.^4^ Therefore, the assessment of the lumbar strength can be used to avoid primary and secondary traumas.^5^ At present, different valid methods are being used to test the strength of lumbar muscles.^6^ Biering-Sorensen test^7^, is a valid measuring method for the strength of lumbar extensors and a prone bridging test which has shown to be accurate to assess the strength of lumbar muscles.^4^

The tools that can be used for measuring muscle strength are, hand held dynamometry (HHD), Isokinetic dynamometers or Manual Muscle Testing. Handheld dynamometer can ensure quantified strength measurement and clinically, it is very effective and efficient tool.^8^ It is also considered as a reliable and valid tool to measure the strength of muscles in the upper and lower extremities. No functional measuring tool for calculating the strength of lumbar flexors and extensors has been reported. There was also no evidence for HHD as an effective tool to reliably measure the strength of lumbar flexors and extensors. Therefore, the aim of this study was to determine intra-rater and inter-rater reliability of hand-held dynamometer for assessing isometric lumbar muscles strength in asymptomatic healthy population.

## METHODS

This cross-sectional study was a part of PhD Physical Therapy project conducted at Department of Physiotherapy, University of Lahore Teaching Hospital, Lahore, Pakistan, from July 2020 to August 2020 by using non probability-purposive sampling technique. After approval from the Institutional Review Board (IRB-UOL-FAHS/690/2020, Dated: 22-01-2020) of the University of Lahore, this study was registered in the ClinicalTrials.gov (NCT04578587).

Thirty healthy subjects (26 males & 4 females) aged between twenty-one to twenty-five years were recruited after their written informed consent for this study. Subjects with the history of acute, subacute or chronic low back pain, history of trauma, neurological disorders, spinal surgery, lumbar spine pathology were excluded from the study. Micro Force Evaluation and Testing-2 HHD (Hoggan Scientific LLC, Salt Lake City) was tested to determine the protocol’s for intra and inter-rater reliability. Both raters were physiotherapists having clinical experience of more than eight years with expertise in assessing isometric muscles strength using HHD. Both raters were unaware of each other’s findings and subjects too were unaware of the results.

Based on the recent evidence, positioning protocol of HHD for lumbar flexor and extensor isometric muscles strength was considered.^2^ The isometric strength of lumbar flexors was assessed in supine position at thirty-degree by placing the dynamometer under the suprasternal notch. Subjects were asked to put their hands over the opposite acromion processes. The isometric strength of the lumbar extensors was assessed in prone position. The dynamometer was placed at the level of T4 and subjects were asked to put their hands on the forehead. A 10 cm belt was wrapped just above the lateral malleolus and another belt over the anterior superior iliac spine (ASIS) to prevent lumbar motion while assessing the strength of lumbar flexors whereas belt was wrapped over the posterior superior iliac spine to measure the strength of lumbar extensors. Subjects were instructed to maximally contract the muscle for five seconds.^9^ Peak force value was recorded in newton (N) and converted into torque values (Nm) by multiplying with moment arm length. Moment arm length was measured between sternum and ASIS for lumbar flexors strength whereas for strength of lumbar flexors the distance between T4 and PSIS was considered. Subjects were reassessed after a week while keeping the same HHD protocol.

Data was analyzed by using SPSS 21. Descriptive statistics of age, height, weight and BMI were calculated. Mean, standard deviation, intra-rater and inter-rater pairwise comparisons and correlations of rater one and rater two for first and second readings were calculated. The intra-rater and inter-rater reliability were calculated by the use of two-way random effects model with multiple raters/measurements reliability (ICC 2, *k*), with 95% confidence interval (CI).^10^ The normality of all data was checked using the Shapiro-Wilk test. Statistically, significant value was agreed at the level of 5%.

## RESULTS

Distribution of each measurement performed by the evaluators at the time one, time two, and their variability are shown in Table-I. Correlation and pairwise comparison is shown in Table-II and Table-III. Agreement for inter-rater and intra-rater reliability was classified as excellent for intra-rater measurements of flexors (ICC = 0.96) and extensors (ICC = 0.97), excellent for inter-rater measurements of flexors (ICC = 0.94) and extensors (ICC = 0.95). Agreement limits for 95% CI are shown in Table-IV and Table-V. The 95% CI agreement limits of flexors for the intra-rater measurements were inferior while superior for extensors to those for the inter-rater measurements.

**Table-I:** Descriptive statistics of age (years), Height (cm), weight (kg) and BMI.

	*Mean*	*S.D*	*Range*	*Minimum*	*Maximum*
Age (years)	22.84	1.21	4	21	25
Height (cm)	174.33	6.83	22.20	163.80	186.00
Weight (kg)	68.58	5.08	17.60	60.40	78.00
BMI	22.52	0.35	1.10	22.00	23.10

S.D: Standard Deviation, BMI: Body Mass Index.

**Table-II:** Intra-Rater pairwise comparison of lumbar flexor and extensor muscle strength of rater 1 and rater 2 for 1^st^ and 2^nd^ reading.

*Flexor*	*Mean*	*S. D*	*Minimum*	*Maximum*	*r (p-value)*	*t-test (p-value)*
Rater 1, 1^st^ reading	142.89	8.32	128.01	156.41	0.995 (<0.001)	19.306 (<0.001)
Rater 1, 2^nd^reading	139.82	8.06	126.90	152.34		
Rater 2, 1^st^ reading	146.78	8.34	131.97	160.37	0.996 (<0.001)	25.795 (<0.001)
Rater 2, 2^nd^ reading	143.40	8.36	128.22	157.62		

*Extensor*	*Mean*	*S. D*	*Minimum*	*Maximum*	*r (p-value)*	*t-test (p-value)*

Rater 1, 1^st^ reading	196.58	9.87	181.97	220.17	0.998 (<0.001)	29.701 (<0.001)
Rater 1, 2^nd^ reading	193.40	10.04	198.93	216.63		
Rater 2, 1^st^ reading	201.05	9.69	186.81	224.59	0.997 (<0.001)	24.956 (<0.001)
Rater 2, 2^nd^ reading	197.73	9.63	182.06	220.88		

S.D: Standard Deviation; r: correlation coefficient, p-value: ≤0.001, t-test: paired sample t-test.

**Table-III:** Inter-Rater pairwise comparison of lumbar flexor and extensor muscle strength of rater 1 and rater 2 for 1^st^ and 2^nd^ reading.

*Flexor*	*Mean*	*S. D*	*Minimum*	*Maximum*	*r (p-value)*	*t-test (p-value)*
Rater 1, 1^st^ reading	142.89	8.32	128.01	156.41	0.999 (<0.001)	-58.400 (<0.001)
Rater 2, 1^st^ reading	146.78	8.34	131.97	160.37		
Rater 1, 2^nd^reading	139.82	8.06	126.90	152.34	0.990 (<0.001)	-16.236 (<0.001)
Rater 2, 2^nd^ reading	143.40	8.36	128.22	157.62		

*Extensor*	*Mean*	*S. D*	*Minimum*	*Maximum*	*r (p-value)*	*t-test (p-value)*

Rater 1, 1^st^ reading	196.58	9.87	181.97	220.17	0.998 (<0.001)	-36.709 (<0.001)
Rater 2, 1^st^ reading	201.05	9.69	186.81	224.59		
Rater 1, 2^nd^ reading	193.40	10.04	198.93	216.63	0.995 (<0.001)	-21.544 (<0.001)
Rater 2, 2^nd^ reading	197.73	9.63	182.06	220.88		

S.D: Standard Deviation; r: correlation coefficient, p-value: ≤0.001, t-test: paired sample t-test.

**Table-IV:** Intra-class Correlation Coefficient for intra-rater reliability.

*Reliability*	*Flexor*	*Intraclass Correlation b*	*95% C.I*		*p-value*

			*Lower Bound*	*Upper Bound*	
	Rater 1, 1^st^ and 2^nd^ reading	0.963^a^	-0.007	0.993	0.000
	Rater 2, 1^st^ and 2^nd^ reading	0.959^a^	-0.002	0.992	0.000

*Intra-Rater*	*Extensor*	*Intraclass Correlation b*	*95% C.I*		*p-value*

			*Lower Bound*	*Lower Bound*	

	Rater 1, 1^st^ and 2^nd^ reading	0.974^a^	0.025	0.995	0.000
	Rater 2, 1^st^ and 2^nd^ reading	0.970^a^	0.012	0.994	0.000

Two-way random effects model where both people effects and measures effects are random.

a The estimator is the same, whether the interaction effect is present or not,

b Type A intraclass correlation coefficients using an absolute agreement definition, p-value: ≤0.001, C.I: Confidence Interval.

**Table-V:** Intra-class Correlation Coefficient for inter-rater reliability.

*Reliability*	*Flexor*	*Intraclass Correlation b*	*95% C.I*		*p-value*

			Lower Bound	Upper Bound	
	Rater 1 and rater 2, 1^st^ reading	0.948	0.012	0.990	0.000
	Rater 1 and rater 2, 2^nd^ reading	0.949	-0.035	0.990	0.000

*Inter-Rater*	*Extensor*	*Intraclass Correlation^b^*	*95% C.I*		*p-value*

			*Lower Bound*	*Lower Bound*	

	Rater 1 and rater 2, 1^st^ reading	.949	.003	.990	.000
	Rater 1 and rater 2, 2^nd^ reading	.951	-.019	.990	.000

Two-way random effects model where both people effects and measures effects are random.

b Type A intraclass correlation coefficients using an absolute agreement definition; p-value: ≤0.001; C.I: Confidence Interval.

## DISCUSSION

This study has focused on exploring whether the handheld dynamometer is a reliable tool for assessing the isometric strength of lumbar flexors and extensors. Intra-rater pairwise comparison and correlation of lumbar flexors strength of rater one and rater two for 1^st^ and 2^nd^ reading was 19.306 (p <0.001) and 0.995 (p <0.001) respectively whereas inter-rater pairwise comparison and correlation of lumbar flexors strength of rater one and rater two for 1^st^ and 2^nd^ reading was -58.400 (p <0.001) and 0.999 (p <0.001). Similarly, inter and intra-rater pairwise comparison and correlation of lumbar extensors strength of rater one and rater two for 1^st^ and 2^nd^ reading was 29.701 (p <0.001), 0.998 (p <0.001) and -36.709 (p<0.001), 0.998 (p<0.001). Previously no researcher had demonstrated the inter and intra-rater pairwise comparison and correlation to find the strength of lumbar flexors and extensors using HHD. Therefore, this was the first study to show intra-rater pairwise comparison and correlation.

Intra-class correlation coefficient for inter rater reliability of lumbar flexors strength was (ICC=0.94-0.94) similar to the study by Harding AT et al which measured back muscle strength using isokinetic dynamometry in two sessions with an interval of seven days on fifty-two healthy subjects. Analysis was done by using (ICC) and had a reliability of 0.83–0.94^11^ A study by A Ilyas et al measured reliability of HHD for assessing isometric shoulder flexor and abductor strength. The HHD showed excellent within day (ICC = 0.99-0.99) and between days (ICC = 0.99-0.99) intra-rater reliability for shoulder flexion and abduction of both sides.^12^Another study by Mentiplay BF et al. examined inter-rater and intra-rater reliability of HHD for assessing isometric muscle strength of lower extremity. Thirty asymptomatic subjects were assessed on two sessions. Inter-rater and intra-rater reliability was moderate to excellent for hip and knee (ICCs ≥ 0.70), poor-good for ankle muscles (ICCs = 0.31–0.79).^13^ Intra-class Correlation Coefficient for intra rater reliability of lumbar flexors strength was (ICC=0.95-0.96) similar to the study of David A. Krause, PT et al and Awatani T et al. which had an excellent reliability (ICC, 0.89-0.95)^14^,^15^ Another study by Ashoi L et al. assessed hip muscle group on seventeen participants. All the measurements were taken by an experienced single rater using HHD with a gap of 30 minutes’ interval. Analysis was done by using intra-tester reliability (ICC_2,1_) with a reliability of 0.85-0.92 for all measurements.^16^ A study by Funny Buckinx et al. measured elbow and ankle muscle groups using HHD with Micro FFT 2 Device. ICC values were 0.60 (0.37–0.83) for the ankle flexors and 0.85 (0.74–0.95) for the elbow flexors^17^ whereas inter and intra-class correlation coefficient for inter rater reliability of lumbar extensors strength was (ICC=0.94-0.95) and (ICC=0.97-0.97) to 0.62 (0.41–0.84) for the ankle extensors to 0.87 (0.79–0.96) for the elbow extensors. 17 A study by Hirano M et al measured isometric knee extensors strength using HHD on forty-two subjects. Analysis was done by using (ICC 1,1) with reliability of 0.75-0.82 respectively^18^,^19^ Another study Tudini F described the reliability of HHD for cervical muscles in healthy adults.

Both intra-rater and inter-rater reliability were excellent (ICCs=0.88 – 0.97) similar to that of our study.^20^ A study by Tarca BD investigated the reliability of HHD for assessing isometric abdominal flexion strength. 35 participants were recruited for test-test reliability on subsequent days. HHD showed good agreement (ICC=0.82) and good consistency (ICC=0.87).^21^ It was observed that all HHD assessments are highly reliable. Thus, HHD is considered suitable for testing the isometric strength of lumbar muscles in clinical setting.

### Limitations of the study

The participants were only tested to measure the isometric strength of lumbar flexors and extensors. Asymptomatic participants were tested by HHD, thus the findings from this study cannot be extrapolated to lumbar symptomatic population.

## CONCLUSION

Based on the results of this study, HHD is a reliable tool for assessing isometric lumbar muscles strength. In a clinical setting, it is viewed as an easy to use, portable and cost-effective for the precise measurement of lumbar muscles strength.

### Author’s Contribution

**FT & SAA:** conceived the idea, designed and are accountable for the accuracy or integrity of the work.

**FT, SAA & AA:** data collection, and manuscript writing.

**SAA & HD:** statistical analysis, and edited manuscript.

**SAA, HD, AA:** reviewed and finally approved manuscript.
